# Dr. Henry Osthues

**Published:** 1915-06

**Authors:** 


**Dr. Henry Osthues,** Niagara 1894, died at his home in Brooklyn, April 4, aged 46, of broncho-pneumonia.

